# MiR-181a regulates lipid metabolism via IDH1

**DOI:** 10.1038/srep08801

**Published:** 2015-03-05

**Authors:** Bo Chu, Ting Wu, Lin Miao, Yide Mei, Mian Wu

**Affiliations:** 1 CAS Key Laboratory of Innate Immunity and Chronic Disease, Innovation Center for Cell Signaling Network, School of Life Sciences, University of Science & Technology of China, Hefei, Anhui 230027, China; 2 Scientific and Educational Department, The second hospital of Anhui Medical University, Hefei, Anhui. 230061, China

## Abstract

Lipid metabolism is important for cellular energy homeostasis. Excessive cellular lipid accumulation is associated with various human diseases such as obesity, cardiovascular disease or even cancer. It has been recognized that miR-181a is an important modulator in regulating T lymphocyte differentiation, vascular development and cerebellar neurodegeneration. Here we reports a novel function of miR-181a in the regulation of lipid metabolism. MiR-181a is able to target isocitrate dehydrogenase 1 (IDH1), a metabolic enzyme in TCA cycle. Via targeting IDH1, miR-181a decreases expression of genes involved in lipid synthesis and increases expression of genes involved in β-oxidation, thereafter inhibiting lipid accumulation. MiR-181a transgenic mice show a lower body weight as compared with their wild-type littermates, and moreover, miR-181a transgenic mice exhibit less lipid accumulation. Collectively, these findings uncover a novel miR-181a-IDH1 axis that has an important role in regulating lipid metabolism, and implicate miR-181a as a potential therapeutic target for lipid metabolism disorders.

Lipid metabolism is a critical process in cellular energy homeostasis, it is important to keep a balance between fat synthesis and fat oxidation. Excessive accumulation of lipid such as triglycerides and cholesterol caused severe diseases, such as obesity, cardiovascular disease and diabetes^1^,^2^,^3^,^4^,^5^.

MicroRNAs (miRNAs) are 18–24 nucleotide long non-coding RNAs that regulate gene expression at the post-transcriptional level. They have been implicated in numerous biological processes, including cell proliferation, cell death, cell differentiation and tumorigenesis^6^,^7^,^8^.

As a single miRNA can simultaneously target multiple genes, hypothetically, miRNA is able to post-transcriptionally regulate expression of genes involved in lipid metabolism at the same time. It has been well known that miRNA plays an important role in the regulation of lipid metabolism. For example, miR-122, a liver abundant miRNA^9^, has been shown to regulate FAS, ACC1, ACC2 to modulate cholesterol synthesis and fatty acid oxidation^10^,^11^,^12^. MiR-33 embedded within SREBF1 genes is a critical regulator in lipid metabolism, since it down-regulates a number of ABC transporters, including ABCA1 and ABCG1, thereby regulating cholesterol and HDL generation^13^. In addition, miR-33 has been proposed in fatty acid degradation and in macrophage response to low-density lipoprotein^7^,^13^,^14^,^15^,^16^,^17^. In this study, we show that miR-181a is able to regulate lipid metabolism through targeting isocitrate dehydrogenase 1 (IDH1). IDH1 has been recently shown to regulate lipid metabolism^18^,^19^. Therefore, our findings uncover a novel and important function of the miR-181a-IDH1 axis in regulating lipid metabolism.

## Results

### MiR-181a down-regulates triglycerides and total cholesterol levels *in vivo*

**Figure 1:**
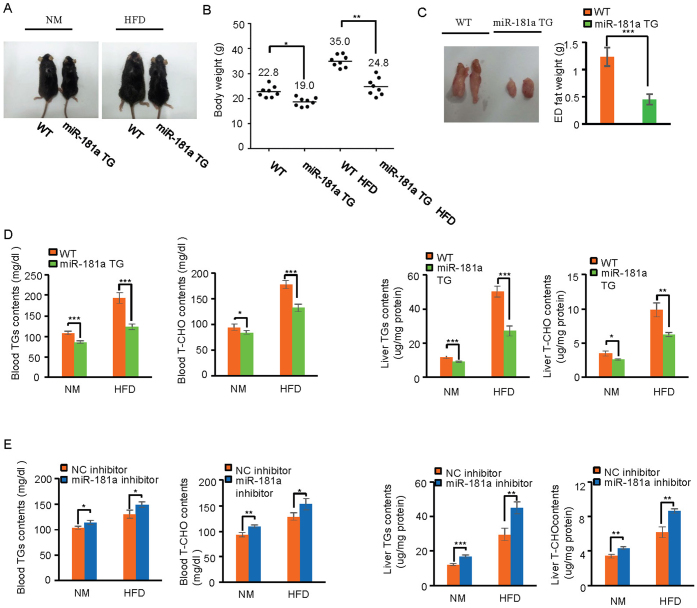
miR-181a reduces triglycerides and total cholesterol levels in mice. (A) MiR-181a transgenic (TG) and wild type (WT) mice were fed with normal diets (ND) or high fat diets (HFD) for 10 weeks. The representative images of miR-181a TG and WT mice were then taken and shown. (B) The body weight of miR-181a TG (n = 8) and WT mice (n = 8) fed with normal or high-fat diet (HFD) for 10 weeks. * and ** indicate p < 0.05 and p < 0.01, respectively. (C) Shown are the representative images of fat from miR-181a TG (n = 6) and WT mice (n = 6) fed with high-fat diet (HFD) for 10 weeks. Fat tissues from these mice were also weighted. The data are statistically analyzed. *** indicates p < 0.001. (D) The blood or liver lysates from miR-181a TG (n = 6) or WT (n = 6) mice feeded with normal diets or high fat diets for 10 weeks were used for TGs and T-CHO levels measurement. *, ** and *** indicate p < 0.05, p < 0.01 and p < 0.001, respectively. (E) MiR-181a WT mice treated with miR-181a (n = 12) or control (n = 12) inhibitors by intraperitoneal injection were fed with normal or high fat diets (HFD) for 4 weeks. The blood or liver lysates from these mice were then used for TGs and T-CHO levels measurement. *, ** and *** indicate p < 0.05, p < 0.01 and p < 0.001, respectively.

We have recently characterized the inhibitory function of miR-181 in the regulation of embryo implantation in mice (unpublished data). Intriguingly, we noticed that miR-181a transgenic (TG) mice exhibited relatively smaller size and lower body weight than miR-181a wild type (WT) mice under normal maintaining conditions (Figure 1A and supplementary Figure S1A). To explore whether miR-181a is involved in the regulation of lipid metabolism, both miR-181 TG and WT mice were fed with high fat diet (HFD), and after 10 weeks of feeding, miR-181a TG mice exhibited smaller size and lower body weight than miR-181a WT mice (Figures 1A and 1B) while these mice showed no obvious differences in food intake (Supplementary Figure S1B). It is worth to mention that miR-181a caused more significant decrease in mice body weight under HFD treatment compared to normal diet treatment (Figure 1B). HFD treatment also led to a decreased accumulation of epididymal white adipose tissue in miR-181a TG mice compared with miR-181a WT mice (Fig. 1C). Moreover, mice treated with miR-181a inhibitor exhibited increased body weight than control inhibitor-treated mice under both normal diet and HFD treatment conditions (Supplementary Figure S1C), and this phenomenon was not caused by changes in food intake (Supplementary Figure S1D). These data strongly suggest miR-181a as a novel regulator of lipid metabolism.

To investigate whether miR-181a could down-regulate levels of triglycerides (TGs) and total cholesterol (T-CHO), liver lysates and blood from miR-181a TG and WT mice were analyzed for TGs and T-CHO contents. TGs and T-CHO levels were decreased in miR-181a TG mice compared to miR-181a WT mice under both normal diet and HFD treatment (Figure 1D). Correlated with more body weight reduction of miR-181a TG mice under HFD treatment, TGs and T-CHO levels were more significantly decreased in miR-181a TG mice when fed with HFD (Figure 1D). We next determined the effect of miR-181a inhibitors on TGs and T-CHO levels in vivo. MiR-181a WT mice were injected with either miR-181a or control inhibitor, and one month later, blood and liver lysates were extracted, TGs and T-CHO were then examined. The results showed that mice injected with miR-181a inhibitors exhibited a great increase in TGs and T-CHO levels when compared with control group (Figure 1E). Taken together, these findings demonstrate the physiological function of miR-181a in decreasing TGs and T-CHO levels.

### MiR-181a inhibits lipid accumulation

**Figure 2:**
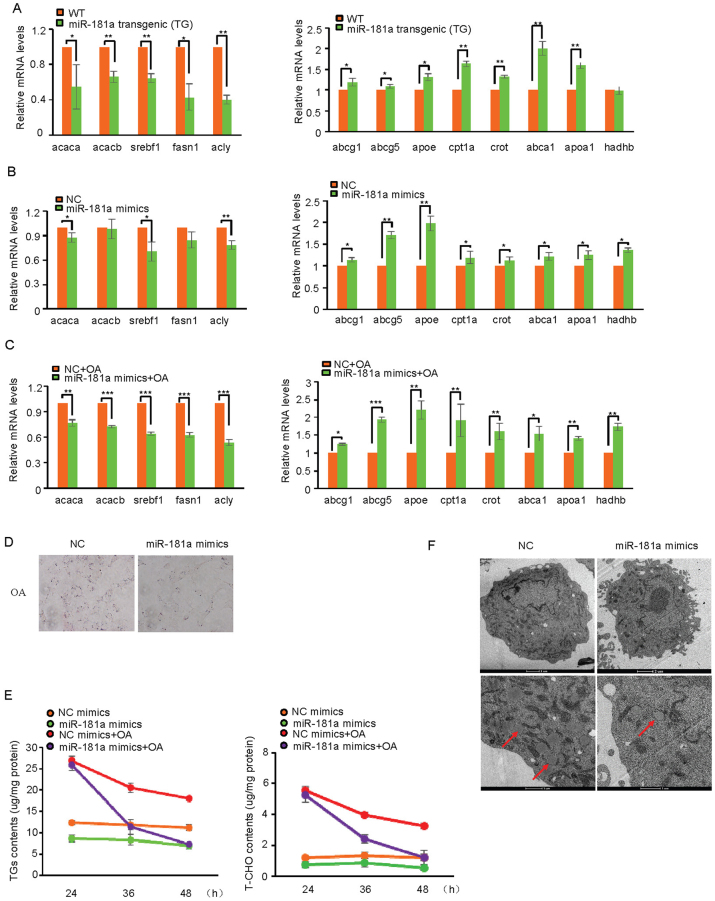
miR-181a inhibits lipid accumulation. (A) Real-time RT-PCR analysis of expression levels of genes involved in lipid synthesis and fatty acid oxidation in livers of miR-181a transgenic and WT mice. Data are mean ± SD from three independent experiments. * and ** indicate p < 0.05 and p < 0.01, respectively. (B) MEF cells were transfected with miR-181a or control mimics. Forty-eight hours later, cell lysates were subjected to real-time RT-PCR analysis to detect mRNA levels of genes involved in lipid synthesis and fatty acid oxidation. Data are mean ± SD from three independent experiments. * and ** indicate p < 0.05 and p < 0.01, respectively. (C) MEF cells were transfected with miR-181a or control mimics. Twenty-four hours later, cells were treated with oleic acid (OA) for another 24 h. Cell lysates were then analyzed by real-time RT-PCR to detect mRNA levels of genes involved in lipid synthesis and fatty acid oxidation. Data are mean ± SD from three independent experiments. *, ** and *** indicate p < 0.05, p < 0.01 and p < 0.001, respectively. (D) MEF cells were transfected with miR-181a or control mimics. Twenty-four hours later, cells were treated with oleic acid (OA) for an additional 24 h. Cells were then stained with Oil Red O (ORO) and visualized under a light microscope. (E) MEF cells were were transfected with miR-181a or control mimics. Twenty-four hours later, cells were treated with oleic acid (OA) for another 24 h. Levels of TGs and T-CHO were measured at the indicated time points. Data are mean ± SD from three independent experiments. (F) MEF cells were transfected with miR-181a or control mimics. Twenty-four hours later, cells were treated with oleic acid (OA) for another 24 h. Cells were then analyzed by electron microscopy. Red arrows indicate the cellular lipid droplets.

To explore the molecular mechanism whereby miR-181a regulates lipid metabolism, we first performed real-time RT-PCR analysis to examine expression levels of the genes involved in lipid synthesis, β-oxidation, or cholesterol transport in livers of miR-181a TG and WT mice. Transgenic expression of miR-181a decreased the levels of the genes involved in lipid synthesis, including Acaca, Acacb, Srebf1, Fasn1 and Acly (Figure 2A). Conversely, the levels of the genes involved in β-oxidation and cholesterol transport were increased upon miR-181a induction, such as AbcG1, Abcg5, Apoe, Cpt1a, Crot, Abca1, Apoa1 and Hadhb (Figure 2A). We next determined the effect of miR-181a on these genes expression in MEF cells treated with or without oleic acids (OA), a well known stimulator of TGs synthesis. The results showed that treatment of miR-181a mimics led to the decreased expression of lipid synthesis-related genes and the increased expression of β-oxidation- and cholesterol transport-related genes in MEF cells treated with and without oleic acids (Figures 2B and 2C). Similar data were also obtained in tail-tip fibroblasts (TTFs) from miR-181a TG mice (Supplementary Figures S2A and S2B). Additionally, miR-181a inhibited lipid levels in MEF cells after OA treatment, as evaluated by Oil Red O (ORO) staining (Figure 2D). Treatment of either miR-181a-overexpressing MEF cells or miR-181a TG mice-derived TTFs with OA showed a much faster decrease in both TGs and T-CHO levels compared to control cells (Figure 2E and supplementary Figure S3). Electron microscopy also revealed that treatment of miR-181a mimics greatly decreased the number of lipid droplets in MEF cells under OA treatment conditions (Figure 2F). Together, these data suggest that miR-181a inhibits lipid accumulation via inhibition of lipid synthesis and stimulation of β-oxidation and cholesterol transport.

### MiR-181a directly targets IDH1 and inhibits its expression

**Figure 3:**
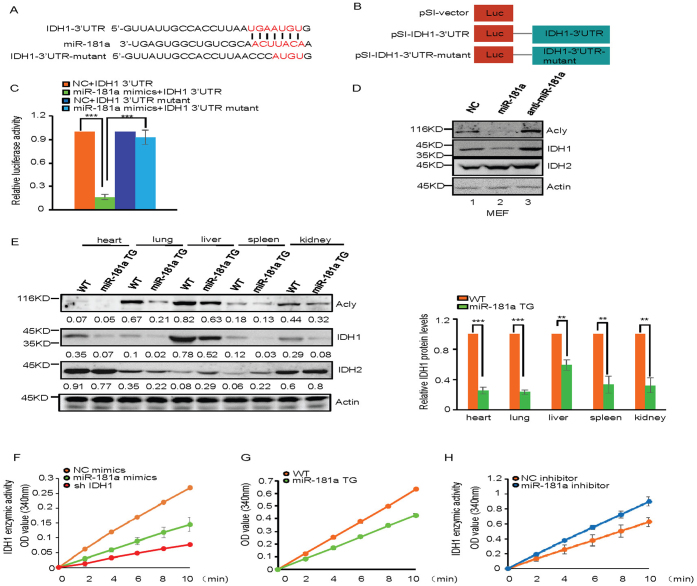
IDH1 is a direct target gene of miR-181a. (A) Illustration of base pairing between miR-181 and the 3′ UTR of IDH1. Substitution of UGA with CCC for the mutant reporter construct is also shown. (B) Schematic illustration of pSI-CHECK based luciferase reporter constructs used for examining the effect of miR-181 on the 3′ UTR of IDH1. (C) MiR-181a mimics were transfected into H1299 cells together with the indicated reporter constructs. Twenty-four hours after transfection, reporter activity was measured and plotted after normalizing with respect to Renilla luciferase activity. Data are mean ± SD from three independent experiments. *** indicates p < 0.001. (D) MEF cells were transfected with control mimics, miR-181a mimics or miR-181a inhibitors as indicated. Forty-eight hours later, cell lysates were analyzed by Western blot with the indicated antibodies. (E) Lysates from the indicated tissues of miR-181 TG and WT mice were analyzed by Western blot with the indicated antibodies. The blots were quantified by using Gel-Pro analyzer software (Rockville, MD, USA). The value of each band indicates the relative expression level after normalizing to the loading control Actin. The ratio of IDH1 to Actin was also calculated. The data are represented as mean ± SD from three independent experiments. ** and *** indicate p < 0.01 and p < 0.001, respectively. (F) MEF cells were treated with control mimics, miR-181a mimics, or IDH1 shRNA as indicated. Forty-eight hours later, cells were subjected to cytosol/mitochondria fractionation. The cytosolic fraction was then assayed for IDH1 enzymatic activity. Data are mean ± SD from three independent experiments. (G) Cells from the livers of miR-181a TG or WT mice were subjected to cytosol/mitochondria fractionation before the cytosolic fraction was assayed for IDH1 enzymatic activity. Data are mean ± SD from three independent experiments. (H) Cells from the livers of miR-181a WT mice treated with miR-181a or control inhibitors by intraperitoneal injection were subjected to cytosol/mitochondria fractionation before the cytosolic fraction was assayed for IDH1 enzymatic activity. Data are mean ± SD from three independent experiments.

To further understand how miR-181a inhibits lipid accumulation, we searched the TargetScan database for the potential targets of miR-181a. Sixteen candidate genes were identified using this method (Supplementary Figure S4A), among which the *IDH1* gene 3′ untranslated region (3′-UTR) contains one putative site (UGAAUGU) that matched to the miR-181a seed region (Figure 3A). IDH1 has been previously reported to regulate lipid metabolism. IDH1 TG mice exhibited abnormal lipid metabolism^19^, which is opposite to the phenotype of miR-181a TG mice, indicating that miR-181a may function through targeting IDH1. To test this possibility, we constructed luciferase reporter plasmids containing either wild type or mutant 3′-UTR of IDH1 (Figure 3B). Induction of miR-181a indeed reduced the luciferase expression from the wild-type but not the mutant reporter plasmid (Figure 3C). We should mention that by using the similar luciferase assay, we did not observe the effect of miR-181a on 3′-UTR of other candidate genes such as LRP4, PPAP and ACSL1 (Supplementary Figure S4B).

To determine whether miR-181a down-regulates IDH1 expression, miR-181a mimics or inhibitors were utilized. Treatment of miR-181a mimics resulted in the reduced protein levels of IDH1 but not its homologous protein IDH2, whereas miR-181a inhibitors showed the opposite effect in MEF cells (Figure 3D). As a control, overexpression of miR-181a decreased, whereas reduced expression of miR-181a increased protein levels of Acly (Figure 3D). Similar results were also obtained in various tissues such as heart, lung, liver, spleen and kidney from miR-181a TG mice (Figure 3E). Correlated with the inhibitory effect of miR-181a on IDH1 expression, both mimics-mediated transient expression and transgenic expression of miR-181a resulted in a dramatic decrease in IDH1 enzymatic activity (Figures 3F and 3G). In contrast, inhibitors-decreased expression of miR-181a greatly increased the enzymatic activity of IDH1 (Figure 3H). These combined data suggest that IDH1 is a direct target of miR-181a.

### MiR-181a regulates lipid metabolism through IDH1

**Figure 4:**
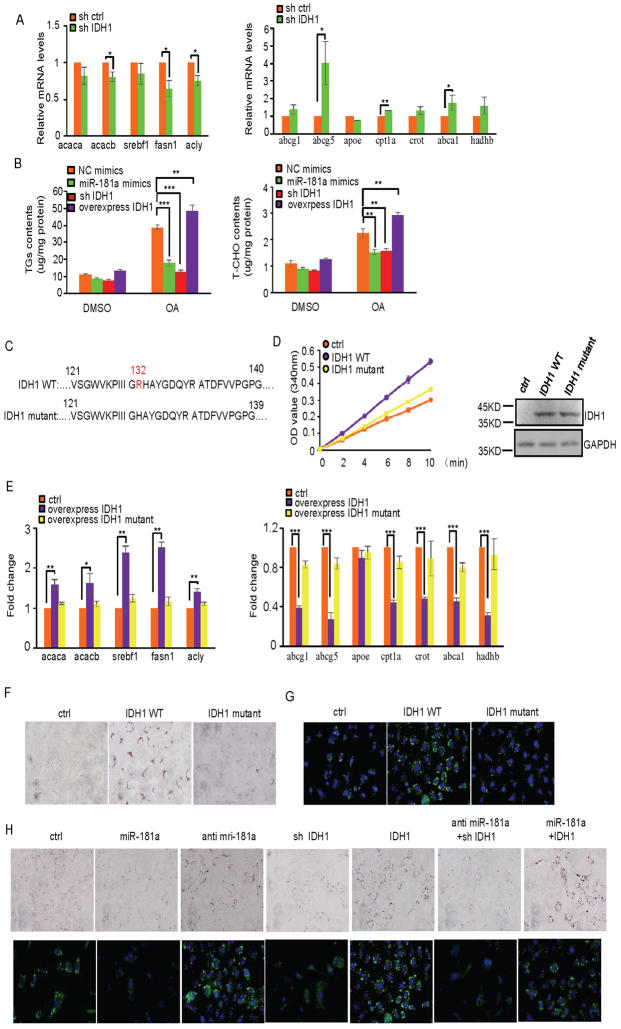
miR-181a inhibits lipid accumulation through IDH1. (A) MEF cells were transfected with control shRNA or IDH1 shRNA. Forty-eight hours after transfection, cell lysates were analyzed by real-time RT-PCR. Data are mean ± SD from three independent experiments. * and ** indicate p < 0.05 and p < 0.01, respectively. (B) MEF cells stably expressing control shRNA or IDH1 shRNA were treated with miR-181a or control mimics as indicated. Forty-eight hours later, cellular levels of triglycerides (TGs) and total cholesterol (T-CHO) were measured. Data are mean ± SD from three independent experiments. ** and *** indicate p < 0.01 and p < 0.001, respectively. (C) Shown are the partial amino acids sequences of wild type and mutant IDH1 proteins. IDH1 mutant lacks Aginine 132 of wild type IDH1. (D) MEF cells were transfected with the indicated constructs. Forty-eight hours after transfection, cells were subjected to cytosol/mitochondria fractionation. The cytosolic fraction was then assayed for IDH1 enzymatic activity. Data are mean ± SD from three independent experiments. Cell lysates were also analyzed by Western blot for wild type and mutant IDH1 expression with anti-IDH1 antibody. (E) MEF cells were transfected with the indicated constructs. Forty-eight hours after transfection, cell lysates were analyzed by real-time RT-PCR analysis to examine expression levels of genes involved in lipid accumulation and fatty acid oxidation. Data are mean ± SD from three independent experiments. *, ** and *** indicate p < 0.05, p < 0.01 and p < 0.001, respectively. (F) MEF cells were transfected with the indicated constructs. Forty-eight hours after transfection, cells were stained with Oil Red O (ORO) and visualized by a light microscope. Shown images are representatives from three independent experiments. (G) MEF cells were transfected with the indicated constructs. Forty-eight hours after transfection, cells were stained with BODIPY and visualized by a fluorescence microscope. Shown images are representatives from three independent experiments. (H) MEF cells were transfected with miRNA-181a mimics, miR-181a inhibitors, IDH1 shRNA, and construct expressing IDH1 protein in the indicated combinations. Forty-eight hours after transfection, cells were stained with Oil Red O (ORO) and BODIPY, followed by visualization with light and fluorescence microscopes, respectively. Shown images are representatives from three independent experiments.

The findings that IDH1 is a direct target of miR-181 and the opposite phenotypes displayed by miR-181a TG and IDH1 TG mice led us to test the possibility that miR-181a may regulate lipid metabolism through IDH1. We first knocked down IDH1 in MEF cells using its specific shRNA. IDH1 knockdown resulted in the decreased expression of lipid synthesis-related genes and the increased expression of β-oxidation- and cholesterol transport-related genes (Figure 4A), which recapitulated the phenotype of miR-181a induction. In addition, induced expression of miR-181a by its mimics or decreased expression of IDH1 by its shRNA markedly reduced levels of TGs and T-CHO in MEF cells (Figure 4B). Conversely, ectopic expression of IDH1 increased cellular levels of TGs and T-CHO (Figure 4B). To further determine whether the enzymatic activity of IDH1 is required for its ability to regulate lipid metabolism, we generated a construct expressing IDH1 mutant where Arginine 132 of wild type IDH1 was deleted (Figure 4C). Compared with wild type IDH1, this IDH1 mutant almost completely lost its enzymatic activity (Figure 4D). Also, in contrast to wild type IDH1, mutant IDH1 failed to regulate lipid levels and expression of lipid synthesis-, β-oxidation- and cholesterol transport-related genes in MEF cells (Figures 4E, 4F and 4G). These results demonstrate that IDH1 regulates lipid metabolism depending on its enzymatic activity. By using ORO and BODIPY staining, we further showed that knockdown of IDH1 in MEF cells reversed the increased levels of lipid levels by miR-181a inhibitors treatment, and induction of IDH1 recovered miR-181a-decreased lipid levels in MEF cells (Figure 4H). These data indicate that miR-181a regulates lipid metabolism through IDH1.

## Discussion

It has been well recognized that miRNAs are involved in the regulation of various cellular activities including lipid metabolism^11^,^12^,^13^,^14^,^15^,^16^,^17^,^20^. MiR-181a has been reported to modulate T cell sensitivity, cell death, cell differatation and tumorigenesis^21^,^22^,^23^,^24^. Here we provide solid evidence demonstrating the critical function of miR-181a in regulating lipid metabolism. Overexpression of miR-181a decreases, whereas inhibition of miR-181a increases IDH1 expression levels and enzymatic activity. More importantly, we show that miR-181a inhibits lipid accumulation through IDH1. Therefore, these data suggest that the miR-181a-IDH1 axis plays an important role in the regulation of lipid metabolism. Our finding indicates the complexity of lipid metabolism, and reinforces the important function of miRNA in the regulation of lipid metabolism.

miRNAs are small RNA molecules with typical length of 18–24 nucleotides that inhibit gene expression via base-pairing with the target mRNAs. It has been well accepted that miRNAs are able to simultaneously target hundreds of different mRNA targets. Therefore, it is not surprised to see that miR-181a is involved in the regulation of various cellular functions. It is possible that miR-181a has diverse functions in different cellular context. It has been shown that miR-181a expression is regulated by a number of signaling networks, including TGF-β, Wnt/β-catenin and STAT3^25^,^26^,^27^. Given the important role of miR-181a in regulating lipid metabolism, it would be interesting to determine whether and how expression of miR-181a is regulated under various lipogenic conditions in the future.

IDH1 functions as a cytoplasmic enzyme (in contrast to IDH2, which locates in mitochondria) serving a significant role in NADPH production. IDH1 has been reported as a potent tumor suppressor in glioblastomas and acute myeloid leukemia^28^,^29^,^30^. Increasing evidence suggests IDH1 as a potential regulator of lipid metabolism. For example, increased expression of IDH1 promotes adipogenesis of 3T3-L1 cells, whereas decreased IDH1 expression inhibits this process. IDH1 transgenic mice exhibit fatty liver, hyperlipidemia and obesity^19^. Additionally, IDH1-dependent reductive glutamine metabolism has been linked to lipogenesis under hypoxia^18^. An association of IDH1 with lipid metabolism is also suggested by the finding that IDH1 gene expression is activated by the transcriptional factors SREBP1 and SREBP2^31^, which are long recognized to play a pivotal role in the regulation of the lipogenic pathways. Here we show that the enzymatic activity of IDH1 is required for its ability to regulate lipid metabolism. Since IDH1 is a major producer of cytoplasmic NADPH, which is absolutely needed for body fat and lipid synthesis, it is conceivable that NADPH may be the downstream mediator of IDH1 in the regulation of lipid metabolism. In support of this idea, IDH1 knockdown markedly decreased levels of NADPH in MEF cells (Supplementary Figure S4C). We also find that similar to induction of miR-181a, knockdown of IDH1 analogously down-regulates the expression levels of lipid synthesis-related genes and up-regulates expression levels of β-oxidation- and cholesterol transport-related genes, thereby inhibiting lipid accumulation. Induction of IDH1, however, shows the opposite effects. These data suggest that IDH1 promotes lipid accumulation by increasing lipid synthesis and decreasing β-oxidation and cholesterol transport, despite the definitive molecular mechanisms that control the expression of genes involved in lipid synthesis, β-oxidation and cholesterol transport by IDH1 remains to be further determined.

In this study, we also observe that miR-181a TG mice exhibit both smaller size and lower body weight than control mice, partially due to decreased levels of triglycerides and total cholesterol. Considering the central role of IDH1 in providing cytoplasmic NADPH, it may not be feasible to consider IDH1 as a target for lipid-lowering strategies. Therefore, our data suggest that miR-181a may be a potential therapeutic target for lipid metabolism disorders such as hyperlipidemia and obesity.

## Methods

### Isolation of tail-tip fibroblasts

To isolate tail-tip fibroblasts, ~2 cm length of tail-tip was cut from two-month-old miR-181a transgenic or wild-type male mice. The epidermis was peeled off and the remaining tissues were cut into 1 mm pieces. The pieces were then transferred to a 6-cm dish and cultured with DMEM containing 10% FBS for one week. Cells migrating out were trypsinized and expanded.

### Cell culture and antibodies

MEF cells were isolated from E13.5 embryos of C57BL/6 mice. MEF cells and tail-tip fibroblasts were cultured with DMEM containing 10% FBS. The following antibodies were obtained from the indicated sources: IDH1 (Santa Cruz 1:1000), IDH2 (Proteintech, 1:1000), Actin (Santa Cruz, 1:1000) and Acly (R&D, 1:1000); HRP-conjugated secondary antibodies against mouse and rabbit IgG (Promega); HRP-conjugated secondary antibodies against rabbit (Promega) and Goat IgG (Santa Cruz).

### Induction of miRNA mimics and inhibitors

MiR-181a mimics and inhibitors were purchase from Genepharma Company (Shanghai, China). The sequences for miRNA-181a mimics and inhibitors were as follows: miR-181a mimics, sense 5′-AACAUUCAACGCUGUCGGUGAGU-3′ and antisense 5′-UCACCGACAGCGUUGAAUGUUUU-3′; and miR-181a inhibitor, 5′-ACUCACCGACAGCGUUGAAUGUU-3′. Transfection of MEF cells by Oligofectamine (Invitrogen) was performed according to the manufacturer's instruction.

### Real-Time RT-PCR

Total RNA was isolated using Trizol (Invitrogen). cDNA was synthesized using PrimeScript RT regent kit (Takara) according to the manufacturer's instruction. The reverse primer for miR-181a were 5′-GTCGTATCCAGTGCGTGTCGTGGAGTCGGCAATTGCACTGGATACGACACTCAC-3′. Real-time RCR was performed using SYBR premix EX Taq (TaKaRa) and ROX and analyzed with Stratagene Mx3000p (Agilent Technologies). Real-time primers sequences were as follows: miR-181a, **5**′-GCGGCAACATTCAACGCTGTCGGTGAGT-3′ and 5′-GTCGTATCCAGTGCGTGTCGTGGAGTCGGCAATTG-3′; Acaca, 5′-TTTCACTGTGGCTTCTCCAG-3′ and 5′-TGCATTTCACTGCTGCAATA-3′; Acacb, 5′-AGGTTCCAGATGCTAATGGG-3′ and 5′-CCCAGGATAAAGCTGGTCAT-3′; Acly, 5′-ACCAGAAGGGAGTGACCATC-3′ and 5′-GATGTTGTCCAGCATTCCAC-3′; Srebf1, 5′-AGCAGGAGAACCTGACCCTA-3′ and 5′-TTTCATGCCCTCCATAGACA-3′; Fasn 1, 5′-TGAGATCCCAGCACTTCTTG-3′ and 5′-TGACATGAACATTGGAGCCT-3′; Abcg1, 5′-CCAGTTCTGCATCCTCTTCA-3′ and 5′-CTCAGGACCTTCTTGGCTTC-3′; Abcg 5, 5′-TCTGTTTCCCATGCTGAGAG-3′ and 5′-AGCAGCATCTGCCACTTATG-3′; Apoe, 5′-TGGAGGACACTATGACGGAA-3′ and 5′-TTGCGTAGATCCTCCATGTC-3′; Cpt 1a, 5′-GTCAAGCCAGACGAAGAACA-3′ and 5′-CTTCAGCGAGTAGCGCATAG-3′; Abca 1, 5′-CTCAGTTAAGGCTGCTGCTG-3′ and 5′-TCAGGCGTACAGAGATCAGG-3′; Hadhb, 5′-CATTTCTGCTGTCAGGCACT-3′ and 5′-AATATTGGTCCGATGCAACA-3′.

### Measurement of TGs and T-CHO levels

Levels of TGs and T-CHO were examined using determined by kits from Changchun Huili Biotech (China) according to the manufacturer's instruction.

### Lipid staining

Lipid levels were measured using Oil Red O (ORO) and BODIPY staining as previously described^32^. Briefly, cells were fixed with 10% buffered formalin for 10 min, and treated with fresh formalin for 2 hours. After being washed with ddH2O, cells were treated with 60% 1, 2-propanol for 5 min. They were then allowed to dry completely and stained with a filtered Oil Red O solution (0.5% Oil red O in 1, 2-propanol) for 10 min. The cells were mounted with glycerol and the images were acquired with bright-field microscopy (Olympus). BODIPY 493/503 (Invitrogen) was used for BODIPY staining, and the images were acquired with an Olympus DP71X microscope (Olympus).

### Electron microscopy

For ultrastructural analysis of cellular lipid droplets by electron microscopy, MEF cells were fixed in 3% glutaraldehyde in 0.1 M MOPS buffer for 8 h at room temperature, then in 3% glutaraldehyde/1% paraformaldehyde in 0.1 M MOPS buffer for 16 h at 4°C, and postfixed in 1% osmium tetroxide for 1 h at room temperature. The cells were then embedded in spurr's resin at room temperature for 4 h and polymerized at 60°C for 2 days. The blocks were cut into micrometre sections with a diamond knife, picked up on 200 mesh grids, stained and observed according to the standard electron microscopy procedures.

### Measurement of IDH1 enzymatic activity

IDH1 enzymatic activity was measured similarly as previously described^30^. Briefly, cells or livers isolated from the mice were subjected to cytosol/mitochondria fractionation before the cytosolic fraction was assayed for IDH enzymatic activity. To measure IDH1 enzymatic activity, 0.2 mg of cytosolic proteins was added to 200 μl of the solution containing 100 mM Tris-HCl (pH 7.5), 1.3 mM MnCl2, 0.33 mM EDTA, 0.1 mM b-NADP+, and 0.1 mM D-(+)-threo-isocitrate. The absorbance of the reaction mixture at 340 nm from NADPH production was recorded continuously on a spectrophotometer (Beckman). The protein concentrations of the cytosolic fraction were determined by a BCA assay kit (Pierce), and enzyme activities were normalized by protein concentrations. Data are plotted as the means of three replicates.

### Generation of transgenic miR-181mice

MiR-181a DNA was cloned into pCAG plasmid. The pCAG-miR-181a plasmids were injected into the zygote pronucleus after they were linearized by I-CEUI. The zygote pronucleus was then transplanted into the oviducts of the surrogate C57BL/6 female mice. 10 days after the pups were born, mouse-tale genotyping was performed by PCR analysis to confirm the transgenic mice carrying the correct gene of interest using the following primer pairs: 5′ GGCACCTTTTGAAATGTAAT-3′ and 5′-CTGTGTCAAAGAAAAGAATCGG-3′.

### Mouse experiments

To determine whether miR-181a is involved in the regulation of lipid metabolism, six-week old miR-181 TG and WT male mice were fed with high fat diet (HFD) for 10 weeks. To knockdown miR-181a in mice, six-week old miR-181 WT male mice were given administration of nanoparticles packed with either control or miR-181a inhibitors four times at one-week intervals^33^. The sequences for control and miR-181a inhibitors were as follows: control inhibitor, 5′-CAGUACUUUUGUAGUACAA-3′ and miR-181 inhibitor, 5′-ACUCACCGACAGCGUUGAAUGUU-3′.

### Ethics statement

All the mouse studies were carried out in accordance with the approved guidelines. All the experimental protocols were approved by the Animal Research Ethics Committee of the University of Science and Technology of China.

### Statistical analysis

Statistical analysis was carried out using Microsoft Excel software and GraphPad Prism to assess differences between experimental groups. Statistical significance was analyzed by Student's t-test and expressed as a P value. P values lower than 0.05 were considered to be statistical significance.

## Author Contributions

B.C. planned and performed most of the experiments with the help from T.W. and L.M. Y.M. and M.W. coordinated the study, oversaw the results and wrote the manuscript. All authors discussed the results and commented on the manuscript.

## Supplementary Material

Supplementary Information Supplementary Figures and legends
