# Supplementary material for: MiR-181a regulates lipid metabolism via IDH1
Source: Sci Rep. 2015 Mar 5;5:8801. doi: 10.1038/srep08801 (PMC4350072; doi:10.1038/srep08801)

Supplementary Information

MiR-181a regulates lipid metabolism via IDH1

Bo Chu[1], Ting Wu[1], Lin Miao[2,*], Yide Mei[1,*] and Mian Wu[1,*]

[1] CAS Key Laboratory of Innate Immunity and Chronic Disease, Innovation Center for Cell Biology, School of Life Sciences, University of Science & Technology of China, Hefei, Anhui 230027, China

[2] Scientific and Educational Department, The second hospital of Anhui Medical University, Hefei, Anhui, 230061, China

Supplementary figure legends

Figure S1

(A) Real-time RT-PCR analysis of expression levels of miR-181a in livers of miR-181a TG and WT mice. Data are mean±SD from three independent experiments. *** indicates $p<0.001$.

(B) Food intake showed no significant difference between miR-181a TG (n=6) and WT (n=6) mice.

(C) The body weight of mice injected with control inhibitors (n=12) or miR-181a inhibitors (n=12) fed with normal or high-fat diet (HFD) for 4 weeks. ** and *** indicate $p<0.01$ and $p<0.001$, respectively.

(D) Food intake showed no significant difference between mice injected control inhibitors (n=12) and miR-181a inhibitors (n=12).

Figure S2

(A) Real-time RT-PCR analysis of expression levels of genes involved in lipid synthesis and fatty acid oxidation in tail-tip fibroblasts (TTFs) of miR-181a transgenic and WT mice. Data are mean±SD from three independent experiments. * and ** indicate $p<0.05$ and $p<0.01$, respectively.

(B) TTFs were treated with oleic acid (OA) for 24h. Cell lysates were then analyzed by real-time RT-PCR to detect mRNA levels of genes involved in lipid synthesis and fatty acid oxidation. Data are mean±SD from three independent experiments. *, ** and *** indicate $p<0.05$, $p<0.01$ and $p<0.001$, respectively.

Figure S3

TTFs were treated with DMSO or OA for 24h. Cellular levels of triglycerides (TGs) and total cholesterol (T-CHO) were then measured. Data are mean±SD from three independent experiments. * and *** indicate $p<0.05$ and $p<0.001$, respectively.

Figure S4

(A) By searching the TargetScan database, the 3’-UTRs of sixteen genes were found

to contain putative sites that matched to the miR-181a seed region. Shown are these potential miR-181a target genes.

(B) MiR-181a mimics were transfected into H1299 cells together with the indicated reporter constructs. Twenty-four hours after transfection, reporter activity was measured and plotted after normalizing with respect to Renilla luciferase activity. Data are mean±SD from three independent experiments. N.S. indicates no significance.

(C) MEF cells were infected with lentiviruses expressing control shRNA or IDH1 shRNA. Forty-eight hours after infection, cell lysates were collected and NADPH levels were measured using a NADPH assay kit. Data are mean±SD from three independent experiments. * indicates $p<0.05$.

Figure S5. Full-length images of the cropped blots presented in the main figures.

(A) Full-length images of Figure 3D.

(B) Full-length images of Figure 3E.

Supplementary Figure S1

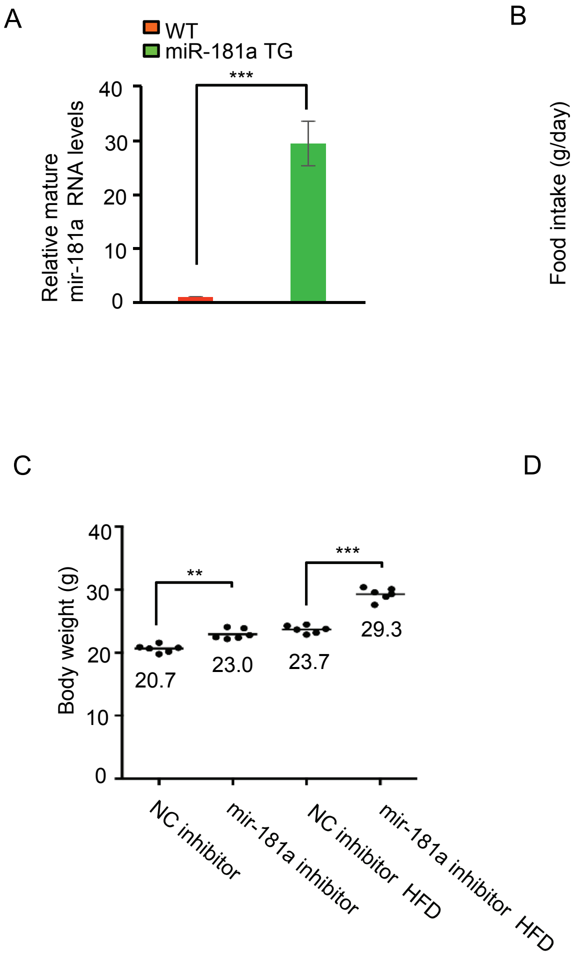

Supplementary Figure S2

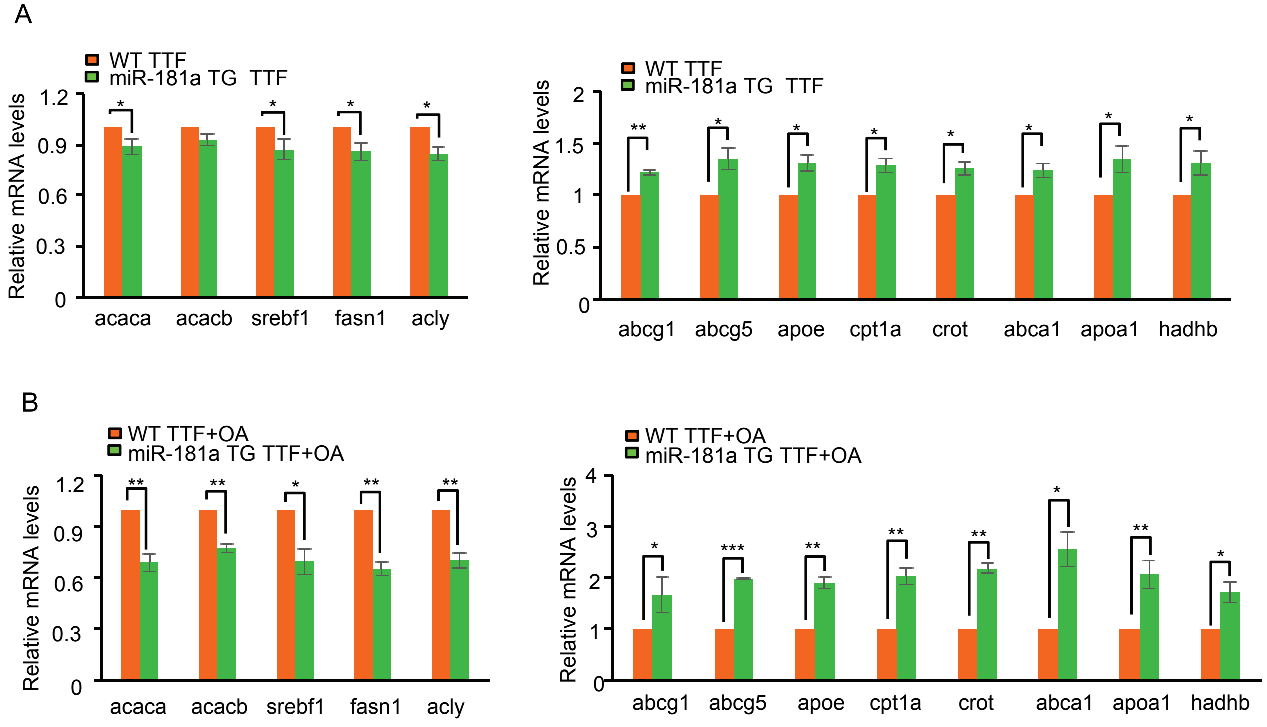

Supplementary Figure S3

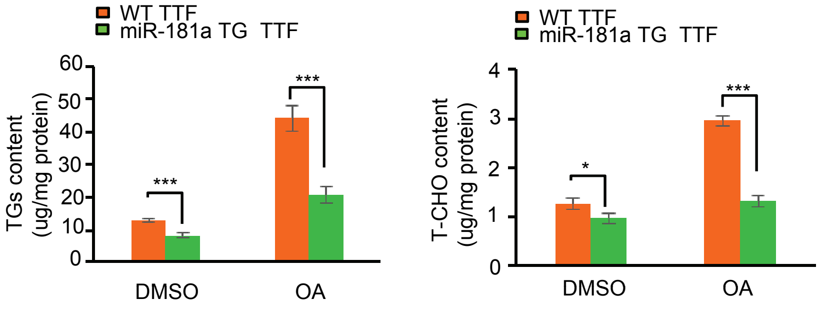

Supplementary Figure S4

A

miR-181a predicted target genes			
IDH1	ACSL1	IRS2	LRP4
SIRT1	TGFBR1	PPAP	HOXA11
LIN28A	MARK1	IGF2BP2	XIAP
GFPT1	CLGALT1	GPD2	IL1A

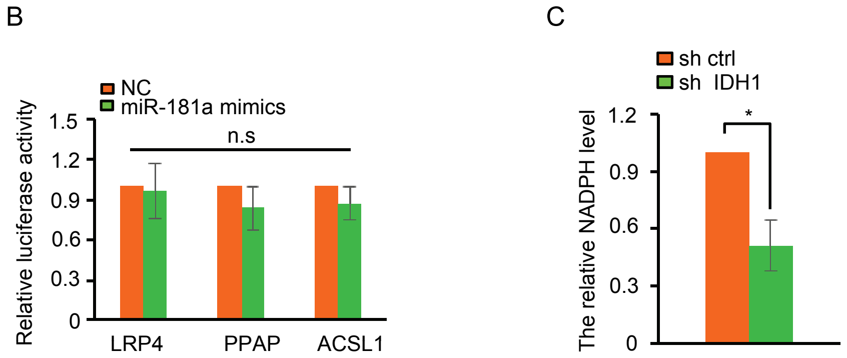

Supplementary Figure S5

A. Figure 3D

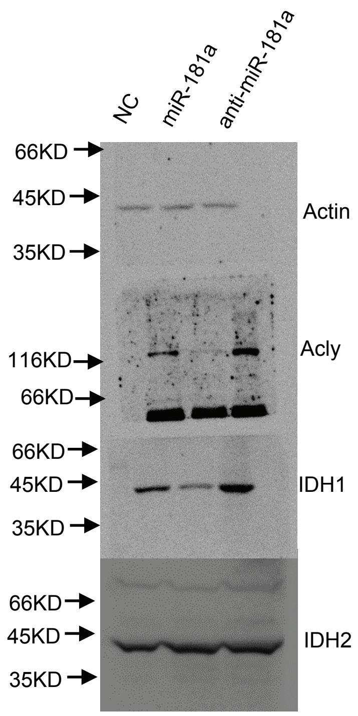

B. Figure 3E

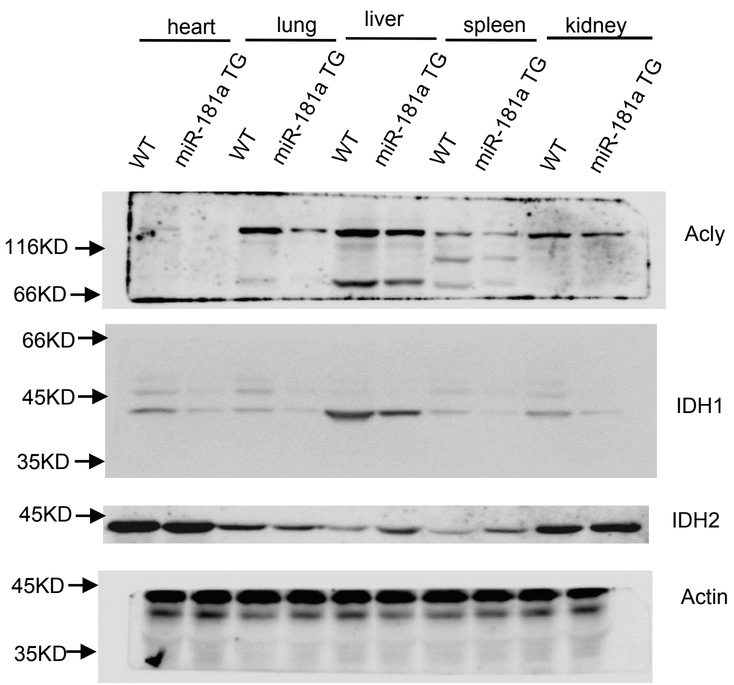

Supplement: Supplementary Information — Supplementary Figures and legends [file srep08801-s1.pdf]
